# Presynaptic Adenosine Receptor-Mediated Regulation of Diverse Thalamocortical Short-Term Plasticity in the Mouse Whisker Pathway

**DOI:** 10.3389/fncir.2016.00009

**Published:** 2016-02-23

**Authors:** Giovanni Ferrati, Francisco J. Martini, Miguel Maravall

**Affiliations:** ^1^Instituto de Neurociencias de Alicante UMH-CSICSant Joan d’Alacant, Spain; ^2^School of Life Sciences, Sussex Neuroscience, University of SussexBrighton, UK

**Keywords:** vibrissae, somatosensory, tactile, whole-cell, short-term plasticity, patch clamp, *in vitro*

## Abstract

Short-term synaptic plasticity (STP) sets the sensitivity of a synapse to incoming activity and determines the temporal patterns that it best transmits. In “driver” thalamocortical (TC) synaptic populations, STP is dominated by depression during stimulation from rest. However, during ongoing stimulation, lemniscal TC connections onto layer 4 neurons in mouse barrel cortex express variable STP. Each synapse responds to input trains with a distinct pattern of depression or facilitation around its mean steady-state response. As a result, in common with other synaptic populations, lemniscal TC synapses express diverse rather than uniform dynamics, allowing for a rich representation of temporally varying stimuli. Here, we show that this STP diversity is regulated presynaptically. Presynaptic adenosine receptors of the A1R type, but not kainate receptors (KARs), modulate STP behavior. Blocking the receptors does not eliminate diversity, indicating that diversity is related to heterogeneous expression of multiple mechanisms in the pathway from presynaptic calcium influx to neurotransmitter release.

## Introduction

Visual, auditory and somatosensory information reaches the neocortex by way of thalamocortical (TC) synapses. Moment-to-moment changes in the size and reliability of these synaptic connections, termed short-term synaptic plasticity (STP), shape the information that reaches the cortex and determine how the response of a thalamic neuron to a sensory event is transmitted. STP acts as a filter of presynaptic patterns, preferentially transmitting particular frequencies or events (e.g., bursts as compared to single spikes; Fortune and Rose, 2001; Abbott and Regehr, 2004; Buonomano and Maass, 2009). When stimulated from rest, “driver” synapses from TC neurons onto excitatory neurons in layer 4 show STP dominated by strong depression (Stratford et al., 1996; Gil et al., 1997, 1999; Chung et al., 2002; Bruno and Sakmann, 2006; Sherman and Guillery, 2006; Lee and Sherman, 2008; Viaene et al., 2011). However, stimulation from rest does not reproduce the physiologically relevant *in vivo* situation in which sensory information does not arrive against a background of perfect silence. Rather, thalamic spiking as delivered to the cortex consists of ongoing sensory and contextual activity (Slézia et al., 2011; Poulet et al., 2012; Ollerenshaw et al., 2014; Bale et al., 2015; Crunelli et al., 2015; McCormick et al., 2015; Urbain et al., 2015). Prior activity sets the amount of TC synaptic depression: active synapses are effectively “pre-depressed” (Castro-Alamancos and Oldford, 2002; Castro-Alamancos, 2004; Boudreau and Ferster, 2005; Reig et al., 2006). The ongoing STP state of synapses determines the regime of operation of the TC network and conditions how information is transmitted (Buonomano and Maass, 2009).

We recently determined the population-level variability of STP in TC connections during ongoing stimulation (Díaz-Quesada et al., 2014). Recording responses of layer 4 excitatory neurons in acute TC slices, we found that although different TC connections share prominent depression during stimulation from rest, STP during ongoing stimulation is highly heterogeneous across connections. Some TC connections are strongly depressing and respond more weakly to shorter inter-stimulus intervals, while others facilitate and show an enhanced response to shorter intervals. A given temporal stimulus pattern can facilitate some synapses while depressing others, implying that different TC synapses are strong at distinct times during ongoing activity. This range of behaviors does not define separate categories of STP; instead, connections form a continuum. STP variability applies across identified spiny stellate neurons, and occurs even for different recordings carried out in the same slice (Díaz-Quesada et al., 2014).

The mechanisms governing the diversity of STP across excitatory lemniscal TC synapses are unknown and could potentially include pre- and postsynaptic loci. Here, we used whole-cell patch clamp recordings in acute slices to uncover mechanisms whose expression covaries with the amount and tendency of STP and localize them pre- or postsynaptically.

## Materials and Methods

### Slice Preparation

All procedures were performed in accordance with national and European Union policies for the care and use of animals in research. The study was approved by the Instituto de Neurociencias and CSIC Ethical Review Committees. TC slices (Agmon and Connors, 1991) were obtained from male and female ICR mice between 14–25 postnatal days of age. This age is later than the established critical period for TC synaptic plasticity (Crair and Malenka, 1995) and the period when sensory responses have been described as facilitating (Borgdorff et al., 2007); over this range of ages, the distribution of STP values does not depend on age (Díaz-Quesada et al., 2014). Slices (350 μm thickness) were prepared with conventional methods (Díaz-Quesada and Maravall, 2008): after killing the animal, the brain was placed in ice-cold cutting solution bubbled with carbogen (95% O_2_, 5% CO_2_) and containing (in mM): 110 Cl-choline, 25 NaHCO_3_, 25 D-glucose, 11.6 Na-aspartate, 7 MgSO_4_, 3.1 Na-pyruvate, 2.5 KCl, 1.25 NaH_2_PO_4_, 0.5 CaCl_2_. The brain was split at the midline and each hemisphere placed on a custom-made wedge at a slope of 50°. Hemispheres were placed lying on their medial face at a tilt of 10° on the sloped surface of the wedge, with the left hemisphere glued onto the right side of the wedge with its rostral edge facing down and the right hemisphere arranged symmetrically. Around three TC slices were collected per hemisphere. Slices were cut on a vibratome (Campden Instruments Integraslice 7550M; Leica VT1000S) and transferred to a chamber containing artificial cerebrospinal fluid (ACSF) continuously perfused with carbogen and incubated at 34°C for ~30 min. They were then kept at room temperature until used. ACSF composition was usually (in mM): 127 NaCl, 25 NaHCO_3_, 25 D-glucose, 2.5 KCl, 1.25 NaH_2_PO_4_, 2 MgCl_2_, 1 CaCl_2_ unless otherwise noted. However, to examine the effects of [Ca^2+^] on STP diversity, the ACSF composition was modified by increasing CaCl_2_ concentration to 2 or 4 mM while reducing [MgCl_2_] to 1 or 0.5 mM respectively. All chemicals were from Sigma-Aldrich unless otherwise noted.

### Recordings

Patch electrodes were pulled from borosilicate glass (1.5 mm outer diameter, 0.86 mm inner; 3–6 MΩ) and filled with internal solution containing (in mM) 130 K-methylsulfonate, 10 Na-phosphocreatine, 10 HEPES, 4 MgCl_2_, 4 Na_2_-ATP, 3 Na-ascorbate, and 0.4 Na_2_-GTP; pH 7.33, 287–303 mOsm. To ensure that measured STP was purely monosynaptic, the internal solution incorporated the intracellular GABA_A_ antagonist dinitrostilbene-2,2′-disulfonic acid (DNDS), a chloride channel blocker (Dudek and Friedlander, 1996; Covic and Sherman, 2011). DNDS (1 mM; Tocris) worked effectively in TC connections (Díaz-Quesada et al., 2014). Kynurenic acid, a blocker of ionotropic glutamate receptors, was tested at various concentrations and found to provide reliable partial blockade at 150 μM. To manipulate adenosine receptor activation, we used the receptor (A1R) agonist adenosine (9-β-D-Ribofuranosyladenine, Adenine riboside, Adenine-9-β-D-ribofuranoside) and two different antagonists, DPCPX and 8-CPT (all from Tocris). For kainate receptor manipulation we used antagonists UBP 310 (Tocris) and NS-102. Recordings were performed at room temperature (24°C). Earlier work found no evidence that temperature (24°C vs. 33°C) influences STP during ongoing stimulation (Díaz-Quesada et al., 2014).

Neurons were selected based on morphological criteria using infrared differential interference contrast optics and patched in the whole-cell mode. Cells with small spherical cell bodies (~10–15 μm in diameter) and dendrites confined to L4, typical of spiny stellate neurons, were chosen. Recordings were not corrected for liquid junction potential. Neuronal responses were measured while stimulating with depolarizing square pulses of 500 ms duration and increasing intensity; only neurons displaying a regular spiking phenotype (McCormick et al., 1985), clearly distinct from fast spiking or low threshold spiking cells, were included in the analyzed data set. Input resistance was 150–500 MΩ and access resistance was under 10% of input resistance; recordings were discarded if access resistance was unstable or the resting membrane potential drifted by more than 10 mV. Data were acquired with an Axon Multiclamp 700-B amplifier (Molecular Devices), filtered at 4–10 kHz, and sampled at 20 kHz (PCI 6040-E; National Instruments) under the control of software custom-written in Matlab (The Mathworks; Pologruto et al., 2003).

### Electrical Stimulation

TC fibers were stimulated with a Pt-Ir concentric bipolar electrode (FHC; outer pole diameter 200 μm, inner pole diameter 25 μm) located in the white matter; a stimulus isolator generated monophasic pulses (Iso Flex; A.M.P.I.). All stimuli were generated in Matlab. To restrict stimulation to a reduced number of fibers (putatively down to a single fiber), we first searched for a stimulus amplitude at which a clear PSP was seen in a fraction of trials. We then further increased amplitude to a level such that PSP size remained stable but each temporally isolated single stimulus evoked a successful response in almost all trials (Díaz-Quesada et al., 2014). This approach ensured that failures of stimulation were negligible, but kept low the number of stimulated fibers. At this stimulation intensity, successful PSPs maintained their stereotypical shape throughout a train of repetitive stimulation, suggesting that the fibers contributing to the response remained stable. Experiments with unstable success probability or response characteristics (latency, shape) were discarded. Stimulus amplitudes were 1–15 μA, towards the lower end of previously reported thresholds for TC activation and an order of magnitude lower than thresholds for antidromic activation of corticothalamic neurons (Rose and Metherate, 2001).

Stimulation protocols were adapted from Díaz-Quesada et al. (2014). In brief, they consisted of sequences of regular and irregular pulse trains. A regular train was followed by an irregular train, both with the same average frequency (4.59 Hz) and duration (21 pulses, 4.36 s). The regular train had a constant ISI and the irregular train consisted of pulses at different interspersed intervals in the range 13–806 ms. A single specific irregular train was used. Each sequence (9 s long) was repeated 10–15 times per recording; each trial lasted 10 s, including periods of silence during which baseline properties were monitored. Additionally, there was a stimulation pause between trials >5 s (trial start corresponded to condition “from rest”). All protocols were applied with the same stimulation intensity.

### Analysis

To compute PSP amplitude, we searched for the first membrane potential peak in the window extending from 0.5 to 12 ms after the stimulation pulse, averaged the membrane potential over five data samples (from −0.1 to 0.1 ms relative to the raw peak), and subtracted a baseline averaged over 2 ms immediately preceding the stimulation pulse. This short baseline effectively compensated for depolarization caused by earlier PSPs. Mean PSP amplitude was computed separately for each stimulus in a train after removing stimulus artifacts with median filtering. The steady-state response level was assessed by discarding the first five PSPs from stimulation onset (i.e., approximately the first second of stimulation), and computing the mean amplitude over all remaining PSPs.

We quantified STP magnitude using two measures, as follows. First, for each connection we constructed a tuning curve, plotting PSP magnitude as a function of the preceding ISI during ongoing irregular stimulation. Tuning curves were constructed only from steady-state PSPs, discarding the first few responses from rest. We computed the slope of the tuning curve by linear regression over the range of intervals up to 218 ms. This tuning curve slope (TCS) provided a simple measure of whether a connection tended to respond more to shorter or to longer ISIs. Connections with smaller responses to short intervals (i.e., to high instantaneous frequencies) had positive TCS, while connections with larger responses to short intervals had negative TCS. This simple quantification of response tuning disregards effects on timescales longer than a single ISI. We also computed each connection’s relative response upon transitioning from stimulation at a constant frequency to irregular stimulation at a higher frequency, hereafter referred to as “facilitation index”. To obtain the facilitation index, we took the ratio of the average PSP amplitude evoked after the first two intervals after the switch from regular to irregular stimulation, to the steady-state PSP amplitude just before the switch. The resulting index was <1 when the mean response amplitude was reduced upon transitioning to higher-frequency irregular stimulation, and >1 when amplitude was increased. The facilitation index was potentially sensitive to timescales longer than a single ISI; its goal was to quantify the degree of context-dependent facilitation or depression during ongoing stimulation.

All analyses were conducted in Matlab (The Mathworks).

## Results

### STP During Ongoing Stimulation Depends on Presynaptic Mechanisms

**Figure 1:**
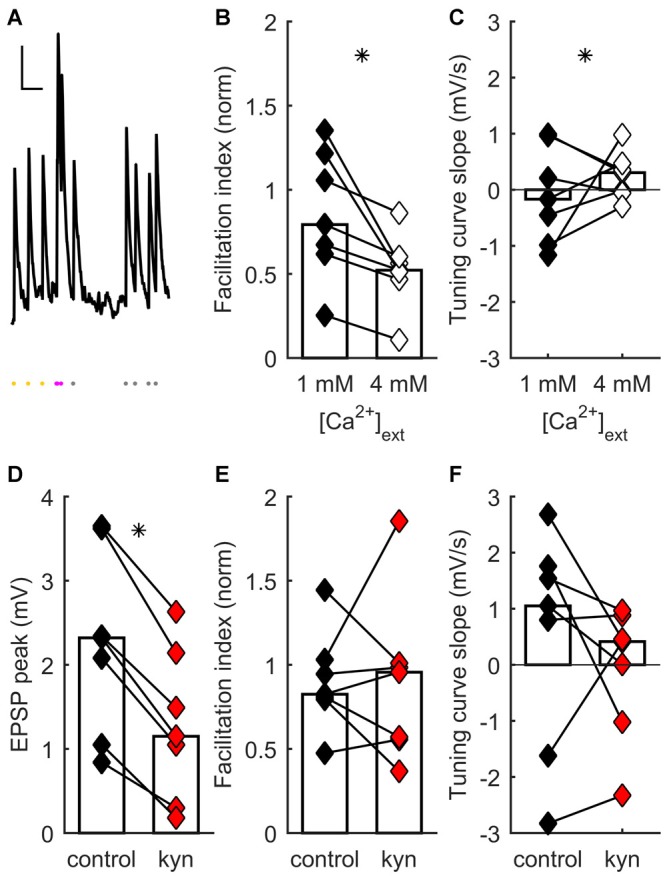
**Short-term synaptic plasticity (STP) diversity depends on presynaptic mechanisms. (A)** Example of recording of post-synaptic potential (PSP) responses to ongoing thalamocortical (TC) stimulation (mean of eight traces). Scale bars: 300 ms, 0.5 mV. Dots at bottom, times of stimulation. Stimulation at constant frequency shown in yellow; stimulation just after transition to irregular train, in magenta; later irregular stimulation, in gray. The facilitation index was obtained by dividing the average response just after switching to irregular stimulation (magenta dots) by the average steady-state response at constant frequency (yellow dots). **(B)** Facilitation index dependance on extracellular [Ca^2+^]. In **(B–E)**, each connected pair of points is one recording. Bars represent population median. Asterisks denote statistically significant difference between distributions (tests are indicated in main text). Facilitation index values decrease and become more narrowly distributed as [Ca^2+^] increases. **(C)** Tuning curve slope (TCS) dependance on extracellular [Ca^2+^]. Distribution becomes narrower as [Ca^2+^] increases. **(D)** Kynurenic acid partial block of postsynaptic glutamate receptors decreases EPSP size. **(E)** Kynurenic acid application has no systematic effect on facilitation index. **(F)** Kynurenic acid has no systematic effect on TCS.

To search for mechanisms regulating differences in STP across TC synapses, we performed whole-cell patch clamp recordings of postsynaptic potentials (PSPs) from visually identified regular spiking neurons located in layer 4 of mouse TC slices (Figure 1A).

The observed diversity of STP across different synapses could result from the differential contribution of disynaptic inhibition to the overall synaptic response. Responses of layer 4 neurons to TC stimulation have a strong disynaptic inhibitory component (Agmon and Connors, 1991; Porter et al., 2001; Gabernet et al., 2005; Wilent and Contreras, 2005; Sun et al., 2006; Cruikshank et al., 2007; Daw et al., 2007). An observed short-term facilitation of the PSP response could potentially arise from faster short-term depression of the inhibitory component relative to the excitatory component (Beierlein et al., 2003; Gabernet et al., 2005; Higley and Contreras, 2006; Heiss et al., 2008). However, in previous work we showed that disynaptic inhibition is not necessary for STP diversity by conducting experiments under intracellular blockade of GABA_A_ receptors: a similar range of behaviors, encompassing depression to facilitation, is found in recordings with intact inhibition and recordings where GABAergic inputs are blocked (Díaz-Quesada et al., 2014). Thus monosynaptic TC connections to cortical layer 4 display STP diversity, which must originate in differences in the properties of those connections.

STP diversity could be pre- or postsynaptically regulated. We conducted a series of experiments to establish the locus of regulation, as follows. Earlier results had shown that extracellular [Ca^2+^] influences STP, because there was significantly less depression at [Ca^2+^] = 1 mM than at 2 mM (Díaz-Quesada et al., 2014). This suggested a presynaptic locus for STP regulation (Zucker and Regehr, 2002; Fioravante and Regehr, 2011). We reasoned that, if the main locus is presynaptic, saturating presynaptic terminals with a much higher extracellular [Ca^2+^] (4 mM) should further bias results towards depression, possibly decreasing STP variability across the recorded population. To test this, we recorded TC synaptic responses in a set of neurons while switching extracellular [Ca^2+^] from 1 to 4 mM (see “Materials and Methods” Section). As expected, increasing [Ca^2+^] to 4 mM induced an increase in onset PSP response (because of an enhanced initial probability of neurotransmitter release) followed by faster depression. This was reflected in a significant change towards lower values of the facilitation index (*p* = 0.016, Wilcoxon signed-rank test, *n* = 7 recordings; Figure 1B). Moreover there was a significant reduction in the heterogeneity of STP across neurons, such that synapses became depressing (*p* = 0.0026, 2-dimensional Kolmogorov-Smirnov two-sample test, *n* = 7; Figures 1B,C). These results show that STP diversity is influenced by [Ca^2+^] in a manner consistent with presynaptic regulation of neurotransmitter release.

As well as presynaptic mechanisms, did postsynaptic mechanisms play a significant role in setting each connection’s effective STP? One such contribution could come from differences across synapses in postsynaptic summation: for example, broader PSPs might lead to greater effective facilitation. However, differences in PSP width do not influence a connection’s facilitation index and thus do not contribute to STP diversity (Díaz-Quesada et al., 2014). We decided to test specifically for an effect of differences in glutamate receptor activation on STP. We reasoned that any effects of NMDA receptor (NMDAR)-mediated summation, or of modulation in postsynaptic receptor activation (e.g., in saturation or desensitization), would be reduced as a result of partial receptor blockade. We thus partially blocked ionotropic glutamate receptors by adding kynurenic acid to the extracellular ACSF (Elmslie and Yoshikami, 1985). At a concentration of 150 μM, kynurenic acid significantly decreased steady-state PSP magnitude (*p* = 0.016, Wilcoxon signed-rank test, *n* = 7; Figure 1D), consistent with a reduced postsynaptic response to neurotransmitter release. However, kynurenic acid had no effect either on facilitation index (*p* = 0.94, Wilcoxon signed-rank test, *n* = 7; Figure 1E) or on TCS (*p* = 0.38, Wilcoxon signed-rank test, *n* = 7; Figure 1F). Thus, STP diversity remained unaffected by manipulation of postsynaptic ionotropic glutamate receptors.

### STP is Regulated by Presynaptic Adenosine Receptor Activation

Which presynaptic mechanisms could modulate STP differentially across synapses? Several mechanisms in the pathway leading from Ca^2+^ entry to neurotransmitter release could potentially contribute. One prominent mechanism modulating synaptic release involves the action of local neurotransmitters through presynaptically expressed receptors (Zucker and Regehr, 2002). We hypothesized that one or several such types of release modulation could contribute to the regulation of STP.

**Figure 2:**
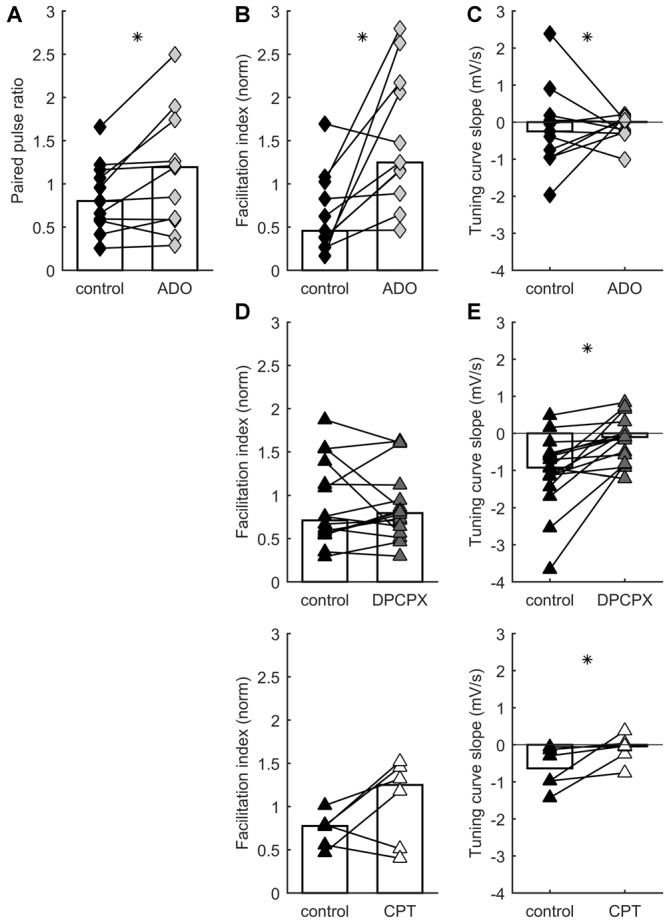
**STP distribution is regulated by A1 adenosine receptor activation.** Each connected pair of points is one recording. Bars represent population median. Asterisks denote statistically significant difference between distributions (tests are indicated in main text). **(A)** Adenosine application increases paired pulse ratio.** (B)** Adenosine application increases facilitation index. **(C)** Adenosine has no systematic effect on TCS. **(D)** A1 receptor blockade by DPCPX (top) or CPT (bottom) has no systematic effect on facilitation index. **(E)** A1 receptor blockade by DPCPX (top) or CPT (bottom) increases TCS.

Adenosine reduces synaptic excitation through the action of presynaptic receptors that inhibit glutamate release (Lupica et al., 1992; Scanziani et al., 1992; Shen and Johnson, 2003; Nicoll and Schmitz, 2005). In TC synapses, application of adenosine decreases EPSCs and increases paired-pulse facilitation (Fontanez and Porter, 2006). To test whether release probability and STP in TC connections can be differentially modulated by adenosine receptors, we recorded responses to TC stimulation before and after adding 100 μM of adenosine to the bath. Consistent with a presynaptic site of action, application of adenosine significantly increased paired-pulse ratios (*p* = 0.024, Wilcoxon signed-rank test, *n* = 11; Figure 2A) but caused no significant change in membrane potential (*p* = 0.24, Wilcoxon signed-rank test, *n* = 11; median depolarization 0.97 mV). Adenosine increased the facilitation index during ongoing stimulation (*p* = 0.0049, Wilcoxon signed-rank test, *n* = 11; Figure 2B), shifting the distribution of STP behaviors expressed in the data set. Median TCS was unchanged (*p* = 0.97, Wilcoxon signed-rank test, *n* = 11; Figure 2C), but variability in this parameter decreased (*p* = 0.0009, *F*-test). This decrease in variability was accounted for by the subset of synapses which, in the absence of added adenosine, had the highest release probability and depressed most strongly: only this minority of synapses had their release probability significantly dampened by adenosine. The dissociation of effects on facilitation index and TCS indicates that adenosine tonically downregulated release probability across the range of intervals (Moore et al., 2003), because the slope relating probability to interval duration was unchanged for the majority of synapses.

Which receptor mediated the modulatory action of adenosine? A1 receptors appear to underpin the inhibitory effects of adenosine on glutamate release (Wu and Saggau, 1994; Dunwiddie and Masino, 2001), including in TC synapses (Fontanez and Porter, 2006). We evaluated the impact of A1 receptors on STP by using the antagonists DPCPX and CPT. We recorded responses to TC stimulation in control ACSF and after addition of 1 μM DPCPX or, in a separate set of experiments, 2 μM CPT. Inhibiting A1 receptors caused a significant change in TCS towards more positive values: i.e., synapses became relatively more responsive to longer rather than shorter intervals (DPCPX: *p* = 0.0011, Wilcoxon signed-rank test, *n* = 16; CPT: *p* = 0.0078, Wilcoxon signed-rank test, *n* = 6; Figure 2E). We interpret this as indicating that A1 receptor inhibition prevented adenosine from limiting glutamate release, leading to a greater tendency towards depression, and more substantial recovery after longer intervals. Conversely, neither DPCPX nor CPT caused a significant difference in facilitation index (DPCPX: *p* = 0.61, Wilcoxon signed-rank test, *n* = 16; CPT: *p* = 0.078, Wilcoxon signed-rank test, *n* = 6; Figure 2D). These effects again shifted the distribution of STP behaviors as compared to control conditions, but did so in the opposite direction to the experiments described above involving application of adenosine. In conclusion, activation of A1 adenosine receptors modulates STP of TC synapses.

### Absence of Evidence for a Role of Kainate Receptors in Regulation of STP

**Figure 3:**
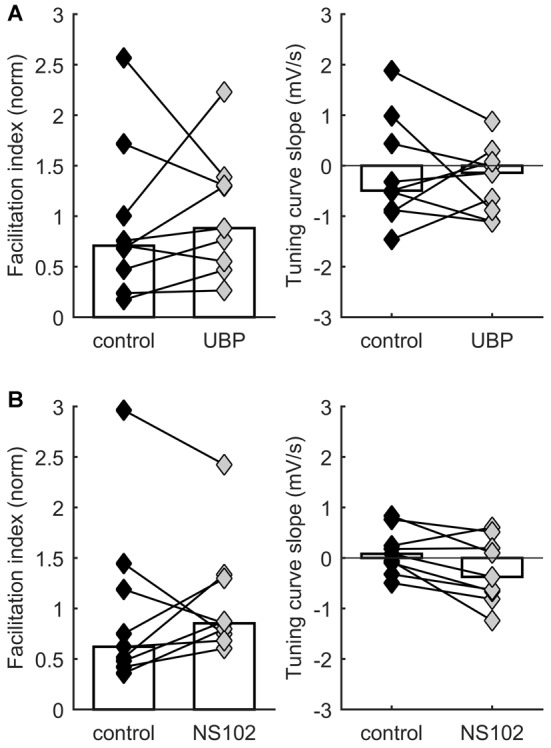
**STP distribution is not consistently affected by kainate receptor activation.** Each connected pair of points is one recording. Bars represent population median. **(A)** No significant effect on facilitation index (left) or on TCS (right) of application of UBP 310, a specific blocker of GluK1-containing receptors. **(B)** No significant effect on facilitation index (left) or on TCS (right) of application of NS-102, a specific blocker of GluK2-containing receptors.

Our experiments demonstrated that activation and manipulation of A1 receptors shifts STP behavior but does not eliminate its diversity. Thus, multiple mechanisms act together to determine STP in each synapse. A possible additional mechanism for regulating STP through neurotransmitter action is modulation by presynaptic KARs. KARs can be powerful presynaptic regulators of synaptic efficacy and STP (Lerma and Marques, 2013). In developing TC synapses, KARs containing GluK1–3 subunits are expressed presynaptically and regulate neurotransmission (Kidd et al., 2002; Urbano and Lerma, 2006; Jouhanneau et al., 2011). We therefore hypothesized that KARs could help modulate release probability and set the level of STP. To test this idea, we compared STP of responses to TC stimulation in layer 4 neurons before and after application of either the selective GluK1 antagonist UBP 310 (10 μM) or the GluK2 antagonist NS-102 (20 μM). We found no consistent effect of UBP 310 across recordings, suggesting that the operation of GluK1 receptors does not systematically modulate STP in TC synapses (*p* = 0.57 for facilitation index, *p* = 0.73 for TCS, Wilcoxon signed-rank test, *n* = 9; Figure 3A). Similarly, we found no consistent effect of NS-102 application (*p* = 0.57 for facilitation index, *p* = 0.054 for TCS, Wilcoxon signed-rank test, *n* = 9; Figure 3B). In conclusion, these experiments found no consistent evidence for a role of these receptor subunits in regulating STP under ongoing stimulation.

## Discussion

Recent work from our laboratory has shown that TC connections do not constitute a population with uniform dynamics. Rather, lemniscal TC connections onto layer 4 spiny stellate neurons respond with diverse STP when stimuli arrive against a background of ongoing activity (Díaz-Quesada et al., 2014). A continuum of STP behaviors is represented across the population. This heterogeneity implies that each synapse is preferentially tuned to certain stimulation intervals, and potentially allows TC pathways to convey rich information about temporal patterns at the population level (Abbott and Regehr, 2004; Buonomano and Maass, 2009; Lee and Buonomano, 2012; David and Shamma, 2013; Chabrol et al., 2015). For example, TC synapses with specific dynamics could act as channels preferentially conveying certain information (e.g., facilitating synapses could act as burst detectors and depressing synapses as “wake-up” signals). Here, we addressed the mechanisms that underpin the diversity of STP. We found evidence for presynaptic regulation of diversity. Modulation of A1 adenosine receptor activation shifts the STP set point.

Many presynaptic mechanisms mediate the complex process from Ca^2+^ entry to exocytosis (Zucker and Regehr, 2002; Fioravante and Regehr, 2011). Diversity of STP within a single population of synapses indicates variability in the expression of this machinery. We found that activation and manipulation of A1 receptors shifts STP behavior but does not eliminate its diversity across the synaptic population (Kerr et al., 2013). This implies that several mechanisms act together to set the overall type and level of STP for each synapse. In our earlier results, the progression of STP during stimulation from rest comprised an initial phase dominated by depression, superimposed with steady-state behavior that could incorporate a varying degree of facilitation (Díaz-Quesada et al., 2014): accounting for this behavior requires phenomena beyond uniform resource depletion (Beck et al., 2005; Kandaswamy et al., 2010; Müller et al., 2010; Hennig, 2013).

We have explored one additional potential mechanism for regulating STP—modulation by KARs. Presynaptically expressed KARs regulate synaptic efficacy (Lerma and Marques, 2013) and are expressed in TC synapses (Kidd et al., 2002; Urbano and Lerma, 2006; Jouhanneau et al., 2011). However, our recordings in the presence of subunit-specific pharmacological blockers of GluK1 and GluK2 failed to find evidence for a role of these receptor subunits in STP under ongoing stimulation. We also performed experiments in slices from knock-out mice for the GluK1 subunit and double knock-outs for the GluK1 and GluK2 subunits (Mulle et al., 1998, 2000; courtesy of Juan Lerma laboratory): these also failed to find a shift in STP behavior across TC synapses (data not shown).

Key questions for future work concern the impact of different mechanisms linking Ca^2+^ entry to triggering of release. STP diversity could result from dynamical regulation of STP at each synapse (Sippy et al., 2003; Cheetham et al., 2007; Branco et al., 2008; Pfister et al., 2010; Carvalho and Buonomano, 2011; Yang and Xu-Friedman, 2012). Multiple steps in the pathway from action potential to exocytosis can be subject to modulation (terminal size; Ca^2+^ influx, distribution and sensing; determination of vesicle availability and fusion; e.g., Sippy et al., 2003; Mochida et al., 2008; Welzel et al., 2011; Zhao et al., 2011; Ermolyuk et al., 2012; Leal et al., 2012; Sylwestrak and Ghosh, 2012; Baden et al., 2014; Fioravante et al., 2014; Calloway et al., 2015; Körber et al., 2015; reviewed in Branco and Staras, 2009; Fioravante and Regehr, 2011; de Jong and Fioravante, 2014). Moreover, additional mechanisms sited postsynaptically as well as presynaptically, and not ruled out by the present study (e.g., metabotropic glutamate receptors, GABA_B_ receptors) could also help tune each connection’s particular STP behavior. Is diversity essentially random (Ribrault et al., 2011), or regulated via modulation of a specific subset of mechanisms? Are these parameters regulated locally at the synapse level or are they set at a cell-wide level (Armbruster and Ryan, 2011; Ermolyuk et al., 2012; Ariel et al., 2013)? Supporting the latter possibility, there is evidence from other pathways that STP properties cluster postsynaptically, i.e., different synapses onto the same neuron can share STP of a similar nature (Branco et al., 2008; Yang and Xu-Friedman, 2012), consistent with postsynaptic regulation by the target cell (Blackman et al., 2013). However, in our present TC data set, preliminary analysis shows no evidence of postsynaptic clustering (data not shown).

## Author Contributions

GF and FJM performed experiments. FJM and MM wrote code for data analysis. GF, FJM and MM analyzed and interpreted the data. MM drafted the article; all authors read and approved the final manuscript.

## Conflict of Interest Statement

The authors declare that the research was conducted in the absence of any commercial or financial relationships that could be construed as a potential conflict of interest.

## Abbreviations

STP: short-term plasticity
TC: thalamocortical
ISI: inter-stimulus interval
PSP: post-synaptic potential
VPM: ventral posterior medial thalamic nucleus
TCS: slope of instantaneous tuning curve
ACSF: artificial cerebrospinal fluid
DNDS: dinitrostilbene-2,2′-disulfonic acid
DPCPX: 8-Cyclopentyl-1,3-dipropylxanthine
CPT or 8-CPT: 8-Cyclopentyl-1,3-dimethylxanthine
UBP 310: (S)-1-(2-Amino-2-carboxyethyl)-3-(2-carboxy-thiophene-3-yl-methyl)-5-methylpyrimidine-2,4-dione
NS-102: 6,7,8,9-Tetrahydro-5-nitro-1H-benz[g]indole-2,3-dione 3-oxime.
